# Exploring the Participation Patterns and Impact of Environment in Preschool Children with ASD

**DOI:** 10.3390/ijerph17165677

**Published:** 2020-08-06

**Authors:** Ghaidaa Khalifa, Peter Rosenbaum, Kathy Georgiades, Eric Duku, Briano Di Rezze

**Affiliations:** 1 School of Rehabilitation Science, McMaster University, Hamilton, ON L8S 1C7, Canada; direzzbm@mcmaster.ca; 2 Department of Paediatrics, McMaster University, Hamilton, ON L8S 1C7, Canada; rosenbau@mcmaster.ca; 3 CanChild Centre for Childhood Disability Research, McMaster University, Hamilton, ON L8S 1C7, Canada; 4 Department of Psychiatry and Behavioural Neurosciences, McMaster University, Hamilton, ON L8N 3K7, Canada; georgik@mcmaster.ca (K.G.); duku@mcmaster.ca (E.D.)

**Keywords:** autism spectrum disorder, participation, environment, social communication, childhood

## Abstract

Participation in everyday activities at home and in the community is essential for children’s development and well-being. Limited information exists about participation patterns of preschool children with autism spectrum disorder (ASD). This study examines these participation patterns in both the home and community, and the extent to which environmental factors and social communication abilities are associated with participation. Fifty-four parents of preschool-aged children with ASD completed the Participation and Environment Measure for Young Children and the Autism Classification System of Functioning: Social Communication. The children had a mean age of 48.9 (8.4) months. Patterns of participation were studied using descriptive statistics, radar graphs, and Spearman correlations. Children with ASD participated in a variety of activities at home and in the community, but showed a higher participation frequency at home. Parents identified different barriers (e.g., social demands) and supports (e.g., attitudes) in both settings. There was a moderate positive association between children’s social communication abilities and their levels of involvement during participation and the diversity of activities. This study highlights the importance of social communication abilities in the participation of preschool children with ASD, and the need to support parents while they work to improve their child’s participation, especially within their communities.

## 1. Introduction

Participation is defined in the WHO’s International Classification of Functioning, Disability, and Health (ICF) as “involvement in a life situation” [1]. Since the introduction of the ICF, this definition has evolved and has been given several meanings in the literature [2,3]. Participation has also been described as the intensity of engagement or being involved in a life situation [2], and as the experience of taking part in an everyday activity [4]. Participation has been defined as a multidimensional concept that includes two essential constructs: *Attendance* to an activity, and level of *involvement* [5,6]. *Attendance* is defined as “being there” and is measured by the frequency and/or diversity of activities in which the person takes part [5]. *Involvement* is defined as “the experience of participation while attending, including elements of motivation, persistence, social connection, and affect” [5]. The definition by Imms and colleagues [5] informed this study, as this multidimensional concept could be applied to any activity or setting, regardless of the ability of the individual [6].

It is believed that participation is a pre-requisite for human development [7] and an indicator of children’s health and well-being [8,9]. According to Bandura’s social learning theory [10], new skills are acquired by direct experience and engagement with and/or through the observation of others. Therefore, through participation in everyday activities, children develop cognitive, sensory, motor, and social skills [11], form friendships, and develop their sense of self-identity [7]. Overall, participation is associated with positive outcomes for all children, but it could have more significant impact on the development of children with disabilities. Participation has been reported to have an influence on learning, independence, and social inclusion of children with disabilities [9].

Over the last decade, the number of children diagnosed with autism spectrum disorder (ASD) has increased, with 1 in 54 children diagnosed with ASD in the US [12] and 1 in 66 in Canada [13]. Children receive an ASD diagnosis during the preschool years (median age of diagnosis is 4 years) [14]. Parents are usually stressed and overwhelmed following receiving an ASD diagnosis [15] and their children’s participation might not be their priority. The preschool years are the period where children first start to learn their roles in a group, gain new skills, and practice these skills in their environments [16]. In addition, participation in the preschool years highly depends on the opportunities offered to children by adults in their everyday environment, typically their parents or caregivers [17,18]. The literature indicates that children with disabilities participate less frequently in domestic, educational, leisure, and social activities when compared to their typically developing peers [11]. Children with ASD are reported to have limited participation, as well as engaging less frequently and in fewer activities when compared to their typically developing peers [19,20]. They participate less frequently in activities of self-care, community mobility, and leisure activities [19]. Families of preschool children with ASD are reported to participate less in special event activities such as family vacations and birthday parties [20]. School-aged children with ASD are also reported to participate less than their typically developed peers in social activities, unstructured activities, and after school activities [21,22].

Many challenges associated with ASD, such as social communication deficits and/or repetitive behaviors, put children with ASD at risk of limited participation. Their social communication difficulties make it a challenge to be involved and engaged with others, which is required for many aspects of participation [19,23]. Furthermore, their restrictive and repetitive behaviors may set them apart from other children and further limit their participation in everyday activities [19]. In addition, parents of children with ASD indicate that their child’s participation may also be impacted because parents may avoid participation outside their home due to fears of the negative perceptions of others [24,25].

As indicated above and in the literature, various aspects of the environment-the physical, social, or attitudinal—can have a significant impact on children’s participation [1,4,26]. Bronfenbrenner’s bioecological systems theory identified the different layers of the environment and their impact on child development [27]. Child development is affected by their interaction with the environment at various levels (directly and indirectly), including their immediate family, community, and society [27]. This emphasizes the need to look at the potential impact of various environments to understand children’s development. Parents of children with disabilities consider the environment to be less supportive and believe that their children have more environmental barriers than typically developing children [9,28]. These barriers could relate to the physical, social, or attitudinal aspects of the environment. The environmental features may either support or hinder the participation of children with ASD. Some of these features include sensory issues, such as level of noise and lighting [29]; furthermore, the physical layout of the space, as well as the social and cognitive demands of some activities, may compromise social connections, such as interacting with others [29]. Availability of resources and services may also support participation of children with ASD [29].

Studies of the impact of the environment on participation with various populations of children with disabilities have shown inconsistent findings. In their study of participation of school-aged children with severe physical disabilities, King et al. [30] found that the environment indirectly impacted on participation. For example, unsupportive environments (e.g., inaccessible or less accommodating) were found to be related to a child’s reduced functional ability and therefore were associated with limited participation [30]. A study of participation of children with cerebral palsy found that environmental factors failed to predict the child’s participation diversity [31]. Moreover, for preschool children with mild developmental disabilities, environmental factors were found to be significant predictors of children’s participation [11]. Studies of participation of school-aged children with ASD found the environment to be one of the factors that impacted their participation [26,28]. In their report, Askari and colleagues [26] reviewed the literature on the impact of the different aspect of the environment (i.e., physical, social, and attitudinal) on participation of children with ASD. In this work, social supports from parents, siblings, or friends were highlighted as important for participation, whereas negative attitudes in the community (e.g., church) presented as barriers for participation for children with ASD [26].

Few measures are designed to assess characteristics of the environment that impact participation in different settings. These include the Child and Family Follow-up Survey (CFFS) [32] and the Participation and Environment Measure (PEM) [33]. The CFFS is designed for children 5 years and older with traumatic brain injuries (TBIs) [32]. It has five sections, one of which is the Child and Adolescent Scale of Participation (CASP), to report on the participation of children with TBIs in the home, school, and community [32]. Another scale is the Child and Adolescent Scale of Environment (CASE), which measures the intensity of the physical, social, and attitudinal environment problems experienced by children with TBIs [32]. The PEM has two versions: The Young Children Participation and Environment Measure (YC-PEM) [34] for children aged 0–5 years, and the Participation and Environment Measure for Children and Youth (PEM-CY) for children and youth aged 5–17 years old [33]. They are used to report on participation and the quality of the environment in various activities in three contexts: At home, daycare/school, and community. PEM can be used with children with various disabilities, as well as children without disabilities.

Another factor that affects participation is the child’s social functioning [35,36]. Social communication functioning is inconsistently defined in the literature [37]. New perspectives in the field are making the distinction between social deficits, impairment, functioning, and abilities [38]. In an extensive search of the literature, King and colleagues developed a conceptual model of factors affecting participation in recreational and leisure activities for children with disabilities [39]; the child’s social functioning was one of the factors identified in this model. Evidence indicated that better-developed social functional ability was associated with better involvement when participating in activities [39]. The Diagnostic and Statistical Manual of Mental Disorders (DSM-5) defines social communication impairment as “deficits in social-emotional reciprocity, non-verbal behavior, and imitative and make-believe play” [40]. Although deficits in social functioning are one of the core symptoms of ASD [39,41], to our knowledge there is a paucity of research on the impact of social functioning on participation for children with ASD.

To date, studies on participation of children with disabilities have focused on school-aged children and adolescents and those with physical disabilities, while there is a lack of research on participation for young children with ASD [24]. A systematic review by Adair et al. [6] found that of the 394 articles on participation that they reviewed, 105 articles focused solely on cerebral palsy, while only 37 articles focused on ASD. Furthermore, these types of studies usually involve comparing a group of children with disabilities to a group of children without disabilities [29,42,43,44,45]. There is a need to study, in depth, the patterns of participation and the potential factors associated with participation amongst preschool children with ASD. The aims of this study were to explore the patterns of participation for preschool children with ASD (3–6 years old) and investigate the impact of different environmental and individual factors on their participation.

## 2. Methods

### 2.1. Participants and Procedures

This cross-sectional study investigated the patterns of participation in preschool children with ASD and the factors that are associated with them, including the environment and the social communication abilities of the child. The study involved analysis of data relating to a subsample of children who were recruited for a larger project (the Pediatric Autism Research Cohort (PARC) project-pilot phase). The subsample included children who have completed the YC-PEM, and therefore involved children who were 5 years and younger. PARC is a longitudinal inception cohort of children recently diagnosed with ASD from Hamilton, Ontario. The study was approved by the local research ethics board (Hamilton Integrated Research Ethics Board (HiREB)) and all families provided informed consent. The sample included 94 children diagnosed with ASD. The inclusion criteria for participants involved being under age 6 at enrollment and being enrolled in services at the regional autism program. 

### 2.2. Assessment Measures

Sociodemographic questionnaire: This questionnaire was created specifically for the PARC study and included questions about the child, such as their age, sex, and country of birth. It also asked questions concerning the family background, including their educational level and family income. 

### 2.3. Participation and the Environment

Participation and Environment Measure (Young children version—YC-PEM): YC-PEM was developed based on ICF concepts and is a parent- or caregiver-completed questionnaire. The current study explored participation at home and community settings only. Home and community are considered the natural learning environment of daily activities for young children [46]. For each activity, parents reported: (i) Frequency of participation on an 8-point scale from never (0) to daily (7) for 13 age-appropriate activity items at home and 11 items in the community; (ii) level of involvement in specified activities (5-point scale from minimally involved (1) to very involved (5)); and (iii) whether caregiver/parent would like to see changes in their child’s participation in this type of activity (yes or no question). For each setting, parents reported on various features of the environment or resources and their impact on their children’s participation, such as the sensory qualities of the environment or the cognitive demands of an activity. For each item in the environment, parents chose one of the following: Whether it has no impact, usually helps, sometimes helps, sometimes makes harder, or usually makes harder. Since the study aim was to explore the pattern of participation, questions on caregivers’ desire to change participation were not considered.

The YC-PEM has shown sound psychometric properties with children with different disabilities. It has an acceptable internal consistency (>0.70) for three scales: Frequency (α = 0.72); Involvement (α = 0.80); and Environmental Support (α = 0.92) [47]. The test–retest reliability for the frequency scale was fair to good for home (ICC = 0.61–0.63) and community (ICC = 0.55–0.63), and for the level of involvement scale reliability was good to excellent for the home (ICC = 0.79–0.93) and good for the community (ICC = 0.71–0.97). The reliability for the environment scale was good for the home and community (ICC = 0.91–0.94) [47]. PEM-CY/YC-PEM has been used to investigate the pattern of participation for children with ASD in different settings, but mostly for school-aged children [29,42,48].

### 2.4. Social Communication Functioning

The construct of social communication was explored using the Autism Classification System of Functioning: Social Communication (ACSF:SC) [49]. The ACSF:SC is a strength-based tool that aims to categorize children with ASD who are between 3 to 6 years old into one of five levels of functioning based on their social communication abilities. This descriptive tool was developed by CanChild researchers based on ICF concepts. The social communication abilities range from level V (lowest ability) through level I (highest ability). This classification tool is not meant to replace any diagnostic or assessment tools, but rather provides a simple standardized method to classify the child’s social communication abilities in a consistent manner among the health provider teams, teachers, and parents [50]. A rater who is familiar with the child is asked to provide two ratings: The child’s capacity level (what the child can do at their best) and the child’s typical performance level (what the child can do on a day-to-day basis). The ACSF:SC demonstrates good intra-rater agreement for parents (k_w_ = 0.61–0.69) and good to very good for professionals (k_w_ = 0.71–0.95) [50]. The inter-rater agreement among parents and professionals ranges from fair to moderate agreement (k_w_ = 0.33–0.53) [50].

### 2.5. Data Analysis

The data for the current analysis were drawn from the initial time point from the larger PARC study. Data were analyzed using STATA software, version 13 (StataCorp LLC, Texas, TX, USA), and an effect was considered statistically significant at α = 0.05.

Descriptive statistics, including the means, standard deviations, and percentages of child characteristics and their family’s sociodemographic information were first calculated for the participants. The distribution of the sample among the five levels of the ACSF:SC was obtained for the best capacity and typical performance scales. To understand the pattern of participation for our sample, the mean and standard deviation were calculated for the frequency and level of involvement scales of the YC-PEM. The percentages of activities in which the children participated were also calculated. Radar graphs were obtained to illustrate the distribution of scores across items. Radar graphs are used to represent the data visually in order to examine patterns of activity and are shaped like histograms. The radar graphs have multiple spokes spreading from the center of the graph, and the longer the spoke, the higher the magnitude of the variable represented by this spoke [51].

To explore the relationships between the ACSF:SC levels and YC-PEM-reported frequency, level of involvement, and the percentage of activity for both settings, scatter plots were created to visualize the data, followed by Spearman’s correlation analysis. The same procedure was done to explore the relationships among the ACSF:SC levels and the environmental scales of the YC-PEM, followed by analysis of variance (ANOVA). ANOVA was conducted to explore whether the size of the differences between best capacity and typical performance levels was associated with the presence of environmental supports or barriers.

## 3. Results

Descriptive statistics: 54 children completed the ACSF:SC and were included in the analysis. Socio-demographic information of the parents, their household, and their child with ASD is summarized in Table 1.

Non-respondent analysis: Six participants did not complete the ACSF:SC and were excluded from the study. There were no significant differences between the respondent and non-respondent children in terms of their age (t (58) = 0.15, *p* = 0.9), gender (Pearson Χ^2^ (1) = 1.0, *p* = 0.3), or language spoken at home (Pearson Χ^2^ (1) = 0.36 *p* = 0.6).

ACSF:SC best capacity and typical performance scores: Parents of 50% of the participants rated their child the same for typical performance and best capacity, and 44.4% of parents judged their children to have lower typical performance abilities than their best capacity ability. Parents of only 5.6% of the participants judged their children to have higher typical performance abilities than their best capacity (Table 2). A total of 46.3% of participants had a ±1-level difference, while only 2% had a 2-level difference.

### YC-PEM

**Figure 1:**
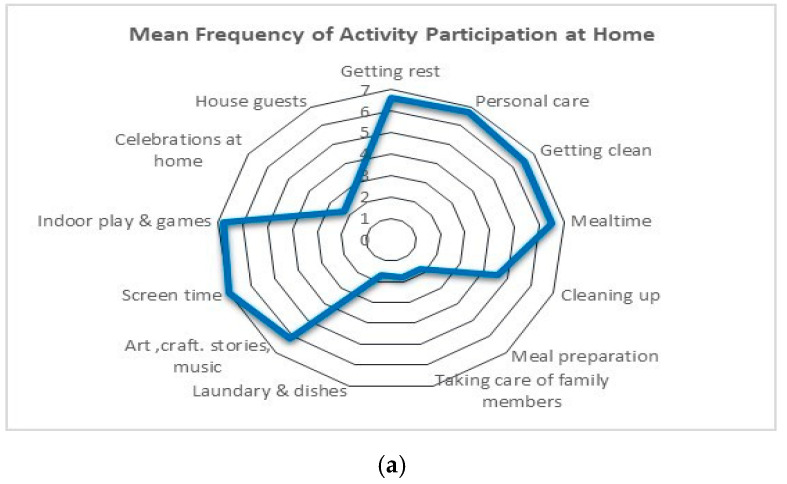

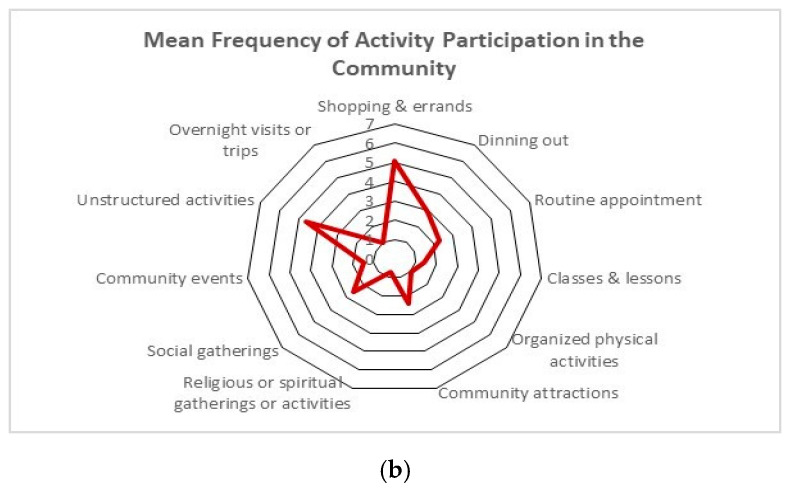
(**a**) Mean frequency of activity participation at home, (**b**) mean frequency of activity participation in the community.

**Figure 2:**
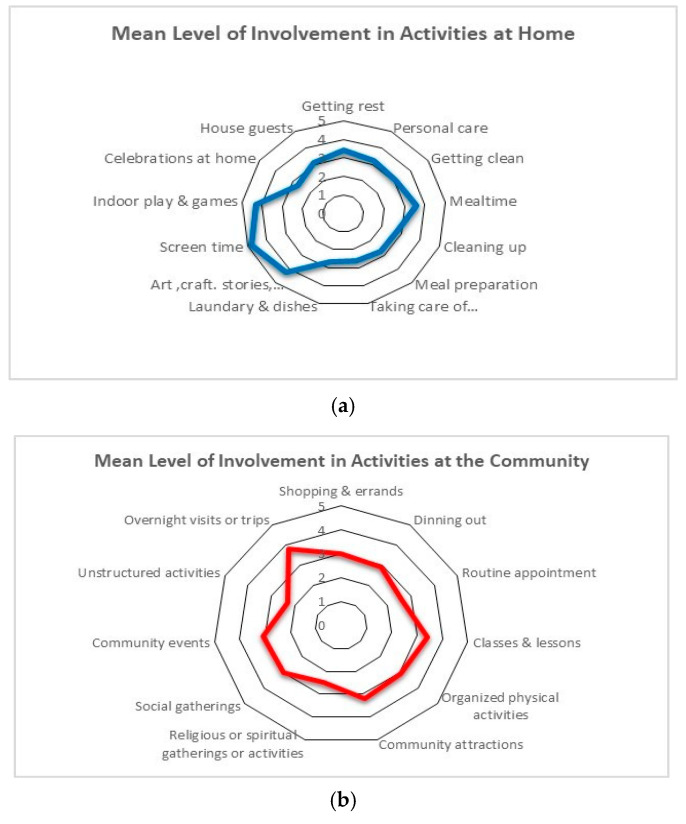
(**a**) Mean level of involvement in activities at home, (**b**) mean level of involvement in activities in the Community.

Participation: Overall, parents reported their children as participating in a variety of activities at home and in the community. Frequency and level of involvement are demonstrated in the radar graphs to depict the activities in which children engaged within the home and community settings (Figure 1 and Figure 2).

## 4. Home Setting

Activity frequency and level of involvement: The majority of our sample (>73%) were reported to participate most frequently in basic care routine activities (mean = 6.6) (Figure 1a); 50% were reported to be “somewhat involved” in these activities (Figure 2a). Household chores were reported to have the lowest frequency in the home setting with a mean of 1.8 (“few times in the last four months”) in three out of four activities. Participants were reported to have different levels of involvement ranging from “not very involved” to “very involved”. Furthermore, up to 65% of the participants were reported to have never participated in these chores. Participants showed high frequency rates in the interactive and organized play with the majority (98%) participating daily in these activities and being very involved. Socializing with friends and family was also reported to have low frequency from “few times in the last four months” (44%) to “a few times a month” (up to 27%). The level of involvement ranged from not very involved to very involved (Supplementary Table S1).

Environmental supports and barriers: Half of the parents reported that the physical layout of their houses supported their children’s participation, as shown in Table 3. Sensory qualities were perceived as a support for 37.7% of the parents. Cognitive and social demands were reported to support children’s participation for 22.6% and 26.4%, respectively, with a further 24.5% and 30.2% parents considering them as barriers. The attitudes of family were reported as supportive for 34.7% of parents. A total of 46.7% of parents considered money and time to support their children’s participation.

Relationship between social communication and participation: Spearman’s correlation analyses provided the same correlations for best capacity and typical performance levels and participation. Therefore, we decided to use the typical performance levels as they represent everyday functional performance. There was very low correlation between participation *frequency* and the ACSF:SC (typical performance level) (r = −0.02, *p* = 0.9). However, the Spearman’s rank correlation showed a low negative correlation between the *level of involvement* and the ACSF:SC (r = −0.32, *p* < 0.01), and a moderate negative correlation between the *percentage of activity participation* and the ACSF:SC (r = −0.42, *p* < 0.01). Because of the scaling of the ACSF:SC, the correlation was negative, but there was a positive association between the level of involvement, percentage of activity participation, and social communication (i.e., the better the social communication ability on the ACSF:SC, the higher the level of involvement and the wider the variety of activities in which the child participates).

## 5. Community Setting

Activity frequency and level of involvement: Overall, the frequency of participation in the community activities was lower than those in the home setting (Figure 1b). Children were reported to participate most frequently in shopping and errands (once a week), followed by unstructured physical activities (a few times a month). The lowest frequency observed was 1 (once in the last four months) for classes and lessons, organized physical activities and overnight trips, vacations and visits. However, even with the low frequency, children were reported as being involved when doing these activities. In all of the activities, there were parents who reported that their children never participated in these activities. For example, 73.4% of parents reported that their children never participated in an organized physical activity.

*Environmental supports and barriers*: The sensory quality of the environment was reported by parents as a barrier for 23.1% of the participants (Table 4). Cognitive and social demands were reported by parents as supportive for 22% and 11.8% of the participants, respectively, while reported as barriers for 24% and 35.3% of the participants, respectively. Parents reported attitudes and relationships with friends to be supportive for 25% and 24% of the participants, respectively. Personal transportation, equipment, and supplies were reported as supports for more than 60% of participants. Time and money to support their children’s participation at the community were also reported by 40% of the parents.

Relationship between social communication level and participation: There was very low correlation between the ACSF:SC (typical performance) level and the participation *frequency* or the percentage of activity participation. There was a moderate negative correlation between the ACSF:SC (typical performance) level and the *level of involvement* (r = −0.41, *p* = < 0.01). Once again, although the correlation was negative, there was a positive association between the level of involvement and social communication abilities (i.e., the better the social communication ability on the ACSF:SC, the higher the level of involvement when children participate in activities).

ACSF:SC and the environmental supports and barriers: The Spearman’s analysis showed very low correlation (r ≤ 0.03) between the ACSF:SC levels and the environmental supports or barriers for both the home and community settings.

The sample was then divided into three groups based on the size of differences between the best capacity and typical performance levels of the ACSF:SC. In group 1, differences were ≤−1, in group 2 there were no differences, and in group 3 the differences were ≥+1. The ANOVA showed no difference between the groups in terms of home environmental support (F (2, 50) = 0.08, *p* = 0.9). Since the environmental barriers at home, as well as environmental supports and barriers in the community were not normally distributed, the Kruskal–Wallis tank test was conducted but no difference was found between groups (*p* > 0.05).

## 6. Discussion

This descriptive study explored participation patterns of preschool children with ASD and factors associated with participation, including the environment and the social communication abilities of the child.

Participation pattern for preschoolers with ASD in different settings: Overall, preschool children with ASD participated in a variety of activities at home. Organized play activities, such as screen time, indoor play, and games, were reported to have the highest frequency, which was also reported in other studies [19,29,42,52]. In fact, in one study, children with ASD had a higher frequency of participation than their typically developed peers in activities such as watching TV and screen time [29]. These activities usually do not involve socializing or engaging with others. Previous studies found that children with ASD usually participate in activities alone or with few people—usually their families [22,42].

Children in this study were also reported to have the lowest frequency of participation in household chores. For example, the mean frequency of participation in meal preparation was 1.8 (out of 7), for taking care of family members was 1.8, and for laundry and dishes was 1.7. These findings were also evident in the literature with preschool and school-aged children with ASD [19,53]. When asked, parents revealed that they did not consider assigning chores to their children with ASD [19]. Parents reported that offering chores to their children with ASD would require a lot of energy to accommodate their children’s behaviors and needs, and therefore they chose not to engage them in these activities [19]. Participating in chores could provide children with ASD with the opportunity to practice their problem-solving skills, increase family socializing, teach them to take responsibilities, and prepare them to take care of themselves and others [54,55].

Our findings also indicated that children with ASD generally have lower rates of participation in community settings (mean = 2.9) when compared to a home setting (mean = 5.9). The same findings were reported for children with various disabilities [56,57]. Parents of children with ASD reported having less control over the environment in the community [11,53]. It is more challenging for parents to manage their children’s behavior in the community due to the unpredictability of the situations and sensory stimulation. As such, families reported that their energy is spent trying to think about the environment—what to expect and how their child may react [19]. The whole process is exhausting for them and consequently they avoid participating in activities in the community [53]. When considering participation for children with ASD in the community, this highlights the importance of taking the whole family into consideration as a unit, rather than only focusing on the child and their capabilities. These findings are consistent with Bronfenbrenner’s Ecological Theory of Human Development [27], in which the child’s developmental outcome is influenced by their interactions with different levels of the environment. At the level of the microsystem, child development is influenced by their immediate environment, which typically includes the family [27]. Parents are responsible for offering opportunities for their children to participate [19,53]. In one study, parents reported avoiding dining out or taking their child to grocery stores because of their risk of a behavioral meltdown [53]. This is supported by our findings that even though these children have generally lower frequency of participation in the community, some were reported to have a high level of involvement of participation in activities in the community. For example, participating in overnight trips and vacations had the lowest frequency (1 out of 7) in the community; however, children who participated had the highest level of involvement (3.8 out of 5) compared to all other community activities. Although there were no control groups in the current study to see how patterns of participation in this cohort compare to those of children without ASD, other studies have reported common findings that children with disabilities have lower participation frequency and involvement than children without disabilities [26,28]. Even when children with and without disabilities participated in the same activities, their levels of involvement are different [26].

Environmental barriers and supports: Parents reported a variety of environmental supports and barriers. However, in some cases what was reported as a support for some parents was considered as a barrier for others. For example, 22% of parents considered the cognitive demands of an activity as a support to their child’s participation at home; however, the same percentage of parents considered it as a barrier. The same applies for social demands of the activity and the availability of services, where similar percentages of parents had considered it as either a support or a barrier. This underscores the importance of taking into consideration the individual variations among children with ASD and how the needs of each child vary in different contexts [28]. Furthermore, sensory qualities of the environment were considered mainly as a support at the home, while a higher percentage considered it as a barrier in the community. This lends further support to the fact that parents’ lack control over the community environment and its impact on their children’s participation. It also supports the findings of another study where atypical sensory processing, such as hyper-responsiveness, was associated with lower frequency of activity participation in the community [58].

Relationship between social communication and participation: Our findings indicated that better social communication abilities were associated with a wider variety of activities in which the child participated at home, and higher levels of involvement when participating in these activities. However, in the community, better social communication abilities were only associated with higher level of involvement, which aligns with the findings that identify the complexity of participation in the community and the different factors that impact it.

### Clinical Implications

The study findings provide some insights for clinicians who work with children with ASD and their families. One important implication for service providers is actively to encourage parents of preschoolers to involve their children in as wide a range of daily activities and recreational opportunities as possible from a very young age, so that “participation” becomes part of daily life and is not then seen as a prescribed “add-on”. Young children’s participation in activities is a reflection of their family choices, available opportunities, as well as their abilities and interest. Whereas typically-developing children often take the initiative to be involved in activities, families of children with ASD need to be supported and encouraged to see opportunities to help improve their children’s participation at home and in the community. Considering each child’s individual needs, clinicians could provide some strategies to improve their participation. For example, household chores could be modified and broken down into several steps that the child could follow to improve various skills, such as their problem-solving skills. Clinicians could also provide some strategies to manage children’s behavior in the community to increase their participation. When recommending interventions, clinicians need to take into consideration the family as a whole and any special situations they might have. Clinicians should also be aware of the community with regard to sensitivities, needs, and vulnerabilities of children with ASD.

## 7. Limitations and Future Research

Results of this study should be considered in light of possible sampling and data limitations, including the small sample size and the study design. Cross sectional data limits our ability to identify whether social communication ability increases participation or whether the opposite is true. However, the PARC study continues to collect longitudinal data, which will provide the opportunity to further examine this in the future. In addition, only families who are enrolled in ASD services were included, which could be a potential source for sample selection bias (access to service bias). For example, children being seen could have complex issues while children with higher cognitive abilities may not be seen within the clinical setting. Furthermore, this study was based on parents’ recall and no direct observation of children’s participation was conducted. Future studies could include a qualitative dimension for a deeper understanding of children’s participation from parents’ perspectives. Other factors that may impact on participation, such as socioeconomic status and maternal education, could also be investigated in future studies. Participation patterns could also be investigated longitudinally in future studies. Simpson and colleagues (2019) studied longitudinally the participation pattern of children with ASD who are transitioning to adolescents (age 9 and 10 years old) [48]. Over three years, they found a trend regarding socializing and participating in physical activities (participation declined as children’s ages increased) [48]. Similar studies with different age groups are essential and would highlight the important factors to consider for intervention planning to improve or maintain their participation in various activities.

## 8. Conclusions

This study adds to the emerging body of literature on participation patterns for preschool-aged children with ASD. In addition, it explores the relationship between social communication and participation, which is a key factor central to ASD. Preschool children with ASD participated in various activities at home and in the community, which are the main environments for participation for this age group. However, parents need support to facilitate and improve their children’s participation in both settings. Furthermore, for interventions to be successful, especially those intended to modify the environment, the individuality of children with ASD, with variable abilities, should be acknowledged and considered when planning intervention goals. In addition, interventions should go beyond modifying the environment around the children and consider the environments that support them, including their family.

## Figures and Tables

**Table 1:** Descriptive statistics and sociodemographic features of the sample.

**Demographic Variables**	**(*n* = 54)**
**Child Gender**	
Boys	45 (83.3%)
Girls	9 (16.7%)
**Child Age** (in months)	
Mean (SD)	48.9 (8.4)
**Language Spoken at Home**	
English	53 (98.2%)
**Caregiver’s Highest Level of Education**	
High School	9 (17%)
Secondary education	44 (83%)
**Spouse Highest Level of Education**	
High School	11 (23.9%)
Secondary education	35 (76.1%)
**Family Annual Income**	
<$30,000	9 (17.6%)
$30,001–$60,000	14 (27.5%)
$60,001–$80,000	5 (9.8%)
>$80,000	23 (45.1%)

**Table 2:** Autism Classification System of Functioning: Social Communication (ACSF:SC) best capacity and typical performance ratings. Agreement between best capacity and typical performance is highlighted.

Best Capacity	Typical Performance					
	I	II	III	IV	V	Total
**I**	2	4	0	0	0	6
	3.7%	7.4%	0.0	0.0	0.0	11.1%
**II**	1	4	7	0	0	12
	1.9%	7.4%	12.9%	0.0	0.0	22.2%
**III**	0	0	10	8	1	19
	0.0	0.0	18.5%	14.8%	1.9%	35.2%
**IV**	0	0	1	6	4	11
	0.0	0.0	1.9%	11.1%	7.4%	20.4%
**V**	0	0	0	1	5	6
	0.0	0.0	0.0	1.9%	9.3%	11.1
**Total**	3	8	18	15	10	54
	5.6%	14.8%	33.3%	27.8%	18.5%	100

**Table 3:** Environmental features as perceived by parents at home.

Environmental Features	Home Setting			
	% Supports	% Barriers	% No Impact	% Sometime Helps/Sometime Make Harder
Physical Layout	55.0	0.0	25.0	21.0
Sensory Qualities	37.7	1.8	37.7	22.6
Physical Demands	30.2	9.4	39.6	20.8
Cognitive Demands	22.6	24.5	20.8	32.1
Social Demands	26.4	30.2	20.8	20.8
Relationships with Family Members	51.9	3.8	5.8	38.5
Attitudes	34.7	4.1	26.5	32.7
Policies	25.5	17.6	35.3	21.6
Services	25.5	27.5	13.7	31.4
Supplies	97.7	0.0	0.0	2.2
Information	50.0	2.3	0.0	47.7
Time	46.7	11.1	0.0	42.2
Money	46.7	4.4	0.0	48.9

**Table 4:** Environmental features as perceived by parents in the community.

Environmental Features	Community Setting			
	% Support	% Barrier	% No Impact	% Sometime Helps/Sometime Make Harder
Physical Layout	30.8	5.8	42.3	21.2
Sensory Qualities	11.5	23.1	19.2	44.2
Physical Demands	19.2	7.8	32.7	38.5
Cognitive Demands	22.0	24.0	16.0	38.0
Social Demands	11.8	35.3	13.7	37.3
Attitudes	25.0	11.5	15.4	48.1
Relationship with Peers	24.0	16.0	18.0	42.0
Weather	7.7	11.5	30.8	50.0
Safety	30.8	9.6	36.5	23.1
Policies	26.0	6.0	34.0	34.0
Personal Transportations	76.5	5.9	11.8	5.9
Public Transportations	21.6	5.9	66.7	5.9
Program & Services	35.3	9.8	5.9	49.0
Equipment or Supplies	86.8	0.0	2.6	10.5
Information	43.6	5.0	0.0	51.3
Time	46.2	7.7	0.0	46.2
Money	43.6	5.0	0.0	51.3

## Supplementary Materials

The following are available online at https://www.mdpi.com/1660-4601/17/16/5677/s1, Table S1: Percentage of children participation frequency and level of involvement.
