# Supplementary material for: Exploring the Participation Patterns and Impact of Environment in Preschool Children with ASD
Source: Int J Environ Res Public Health. 2020 Aug 6;17(16):5677. doi: 10.3390/ijerph17165677 (PMC7460096; doi:10.3390/ijerph17165677)
Supplement: Supplementary file 1 [file ijerph-17-05677-s001.pdf]

International Journal of
Environmental Research and Public Health

Table S1. Percentage of children's participation frequency and level of involvement.

Type of Activity			Frequency	Level of Involvement
Home	Basic care routines	Getting rest	82.7% participate daily	56% somewhat involved<br>30% very involved
		Personal care management	86.5% participate daily	56.9% somewhat involved<br>19.6% very involved
		Getting clean	73.1% participate daily<br>21.1% a few times a week	18% not very involved<br>52% somewhat involved<br>18% very involved
		Mealtime	84.6% participate daily	44.9% somewhat involved<br>36.7% very involved
	Household chores	Cleaning up	15.4% never participated<br>38.5% a few times a week<br>21.2% participate daily	20.9% not very involved<br>55.8% somewhat involved<br>11.6% very involved
		Meal preparation	58.8% never participated<br>11.8% once a week	39.3% not very involved<br>21.4% somewhat involved<br>28.6% very involved
		Taking care of other family members	64.7% never participated<br>11.8% once a week	36% not very involved<br>40% somewhat involved
		Laundry & dishes	62.8% never participated<br>11.8% once a week	38.5%not very involved<br>30.8% very involved
	Interactive & organized play	Art, craft, stories, music	63.5% daily	26% somewhat involved<br>62% very involved
		Screen time	98%daily	88.2% very involved
		Indoor play & games	88.5% daily	30.8%somewhat involved<br>65.4% very involved
	Socializing with friends & family	Celebrations at home	44.2% a few times in the last four months<br>19.2%few times in the last month	31.4% not very involved<br>37.3% somewhat involved

		Houseguests	19.2% never participated<br>26.9% a few times in the last month	23.9% not very involved<br>37% somewhat involved<br>26.1% very involved
**Community**	Neighborhood & community outings	Shopping & errands	32.1% once a week<br>34% a few times a week	22% not very involved<br>56% somewhat involved<br>16% very involved
		Dining out	20.8% never<br>24.5% a few times in the last month	25.6% not very involved<br>51.2% somewhat involved
		Routine appointments	7.5% never<br>24.5%once in the last four months<br>26.4%few times in the last four months	35.4% not very involved<br>45.8% somewhat involved
	Classes & groups	Classes & lessons	64.7%never<br>13.7%once a week	42.9% somewhat involved<br>33.3% very involved
		Organized physical activities	73.4%never<br>12.2%once a week	27.8% not very involved<br>44.4%very involved
	Community-sponsored activities	Community attractions	21.2%never<br>21.2%few times in the last four months<br>17.3%once in the last month	48.7% somewhat involved<br>20.5% very involved
		Religious or spiritual gatherings or activities	82.7%never<br>9.6%once a week	38.5% not very involved<br>30.8% somewhat involved<br>23.1%very involved
		Social gatherings	7.8%never<br>29.4% a few times in the last four months<br>25.5% a few times in the last month	27.5% not very involved<br>40% somewhat involved<br>27.5% very involved
		Community events	29.4% never<br>23.5%once in the last four months<br>21.6%few times in the last four months	26.5% not very involved<br>35.3% somewhat involved<br>26.5% very involved
	Recreational activities & trips	Unstructured activities	26.9%few times in the last month<br>32.7%few times a week	32.7%somewhat involved<br>65.3%very involved
		Overnight visits or trips	42.3% never	42.9%somewhat involved

		32.7%once in the last four months<br>13.5% a few times in the last four months	39.3%very involved
